# Cardiac Autonomic and Salivary Responses to a Repeated Training Bout in Elite Swimmers

**DOI:** 10.3390/sports4010013

**Published:** 2016-02-24

**Authors:** Rohan Edmonds, Anthony Leicht, Brendan Burkett, Mark McKean

**Affiliations:** 1 School of Health and Sport Sciences, University of the Sunshine Coast, Queensland 4558, Australia; redmonds@skidmore.edu (R.E.); mm@markmckean.com (M.M.); 2 College of Healthcare Sciences, James Cook University, Queensland 4811, Australia; anthony.leicht@jcu.edu.au

**Keywords:** heart rate variability, Paralympics, alpha-amylase, athlete, stress

## Abstract

This study examined the acute training responses of heart rate variability (HRV) and salivary biomarkers (immunoglobulin A and alpha-amylase) following a standardised training bout in Paralympic swimmers. Changes in HRV, sIgA and sAA were documented Monday morning, Monday afternoon and Tuesday morning over a 14-week monitoring period leading into international competition. Magnitude based inferences with effect sizes (ES) were used to assess the practical significance of changes each week. Normal training responses elicited increases in HR, α1, sAA and sIgA, accompanied by decreases in HF(nu), standard deviation of instantaneous RR variability (SD1) and the root mean square of successive differences (RMSSD) from Monday morning to Monday afternoon, and to Tuesday morning with similar week to week responses for most variables. Changes in RMSSD from Monday a.m. to p.m. were likely smaller (less negative) for Week 7 (78/18/3, ES = 0.40) following a competition weekend with similar changes observed from Monday a.m. to Tuesday a.m. (90/5/5, ES = 1.30). In contrast, the change in sAA from Monday a.m. to p.m. was very likely less (more negative) at Week 7 (0/0/99, ES = −2.46), with similar changes observed from Monday a.m. to Tuesday a.m. (0/0/99, ES = −4.69). During the taper period, there were also likely increases in parasympathetic modulations (RMSSD, Weeks 12–14) along with increased immune function (sIgA, Week 13) that demonstrated a favourable state of athlete preparedness. Used together, HRV and sAA provide coaches with valuable information regarding physiological changes in response to training and competition.

## 1. Introduction

Understanding the effect of a given training stimulus on exercise performance is critical for planning training sessions and achieving peak performance. Coaches are constantly working to identify how individual athletes respond to training so they can accurately prescribe an appropriate training load. This ideal load reduces the possibility of negative training responses and enhances positive training adaptations. A range of specific blood variables [1] and psychometric questionnaires [2] have been used to monitor an athlete’s response to training loads and quantify athlete fatigue. However, obtaining blood samples can be inconvenient and sometimes painful, especially if used for frequent monitoring. Further, questionnaires can be time consuming and report a subjective measure of fatigue or training load and not internal training load [3]. As a result, there has been increased interest in the monitoring of the autonomic nervous system (ANS) via measures of heart rate (HR), particularly heart rate variability (HRV) [4,5].

Measures of HRV are relatively inexpensive, non-invasive, time efficient and may serve as an important tool for elite athlete populations. With individual changes in cardiac parasympathetic activity proposed as key predictors of improvements in performance [6], the incidence of HRV monitoring of elite athletes has increased due to its ability to document cardiac autonomic responses to training. Understanding the acute HRV responses to regular exercise may help identify ongoing positive or negative training adaptations, allowing coaches to adjust training and recovery protocols with greater accuracy. As a well-recognised marker for assessing training responses in athlete populations, and a marker of predominately cardiac autonomic activity [7], HRV is an important tool used in combination with other physiological markers to comprehensively evaluate an athlete’s training status. While HRV has provided guidance with training adaptations [8] the optimal HRV measure and timing of assessment remains unknown with several studies documenting minimal to no change in HRV with regular exercise/training [5,6]. Therefore, HRV measures may be of partial benefit for practical training guidance, especially during complex training programs or with the elite. Use of other markers in combination with HRV may provide a thorough examination of training adaptations and potentially lead to better guidance of training programs for athletes and coaches.

With recent updates in technology, point-of-care analysis of specific salivary biomarkers is now available, providing coaches with additional viable options when assessing recovery and training responses in a quick and easy manner. One such salivary measure is salivary immunoglobulin A (sIgA), a well-recognized indicator of immune function [9]. The majority of immunological studies of elite athletes have concentrated largely on post exercise salivary immune responses [10] with athletes experiencing a transitory decrease in immune function from 3 to 72 h following heavy training or competition [11]. Recently, there has been conjecture as to the influence of exercise on sIgA with studies identifying both increases and decreases [12] in sIgA concentrations following exercise. As proposed by Nieman’s ‘J shaped’ relationship [13] with highly repetitive workloads or strenuous exercise, there is a degree of immunosuppression, which increases the risk of infection for athletes. Therefore, sIgA may help in identifying appropriate immune function and training adaptation, while also highlighting the early signs of illness, a result of excessive workload or insufficient recovery.

In a similar way to sIgA, real-time analysis of salivary alpha-amylase (sAA), a biomarker of stress and sympathetic nervous system activity [14], is now achievable. Used as a marker of neural activity due to its predominant regulation by the sympathetic nervous system, sAA has been successfully applied as an indicator of exercise intensity in well-trained subjects [15]. Previous research has identified significant increases in sAA, ranging from 200% to 265% above baseline following various forms of high intensity exercise and competition [16,17]. However, these responses appear acute in nature with, sAA returning to baseline at 24 h post exercise [16,17]. Subsequently, sAA change has been proposed as an important training response that may provide potential a more precise guide for prescription of training and recovery cycles in athlete populations [18]. While prolonged increases in sympathetic activity were associated with training maladaptation [19,20], further research may extend our understanding of the acute responses of sAA for use during chronic training.

Recognising the importance of acute training responses and their potential to guide training loads, and avoid training maladaptation, the primary aim of this study was to document the acute responses of HRV, sIgA and sAA to daily activities and a standardised high intensity training bout. Secondly, the effects of chronic training on these acute changes were examined to assist coaches for athlete preparation. It was proposed that using HRV, sIgA and sAA in combination would provide a more holistic assessment for a greater understanding of the acute training responses in athlete populations. This knowledge would then contribute to a more accurate prescription of training and recovery cycles, highlighting the early signs of positive and negative training responses during regular periodised training.

## 2. Materials and Methods

### 2.1. Participant Demographics

Research approval was granted by the local institutional Human Ethics Research Committee with written consent obtained from all athletes before commencement. Athletes from a high performance Paralympic swimming program were recruited for this study with descriptive statistics and impairment type of each athlete shown in Table 1. All swimmers competed in sprint distance events (<200 m), had competed at the national level in the past 12 months and were ranked in the top 10 worldwide for their main event. Each athlete was classified according to the International Paralympic Committee (IPC) swimming classification code and followed periodised training programs prescribed by the head swimming coach (pool sessions) and strength and conditioning coach (strength sessions).

### 2.2. Training Monitoring

Each athlete was monitored during regular periodised training (14 weeks) in the lead up to the 2014 Commonwealth Games and Pan Pacific Para-Swimming Championships [21]. Assessments of resting HR and saliva measures were conducted weekly at 3 time points prior to the start of training (Monday a.m., Monday p.m. and Tuesday a.m.) over the 14-week study period.

#### 2.2.1. Monday a.m. and Tuesday a.m.

Towards the start of each training week, resting HR recordings during the night were acquired (9:00 p.m.–5:00 a.m.) using a Polar RS800CX (Polar Electro Oy, Kuopio, Finland, 1000 Hz). Resting saliva measures were collected at 6:45 a.m. each morning, approximately 10 min before the start of training. These measures were recorded to determine values for HRV, sIgA and sAA prior to the training week and following an afternoon high intensity training session prescribed by the head swimming coach.

#### 2.2.2. Monday p.m.

Resting supine HR and saliva measures were collected at 2:45 p.m. on Monday afternoon, approximately 10 min before the start of training. These measures were recorded to determine values for HRV, sIgA and sAA prior to a high intensity training session prescribed by the head swimming coach. Additionally, these afternoon measures were recorded to examine the influence of daily activity on HRV, sIgA and sAA (*i.e.*, compared to Monday a.m. values).

#### 2.2.3. Training Overview

The training focus of each week varied throughout the 14-week monitoring period with all swimmers following the same training phases that were identified as aerobic maintenance, aerobic capacity, anaerobic capacity, power and speed, and taper (Table 2). As prescribed by the head swimming coach, all athletes trained at a HR below 140 beats per minute (bpm) during the aerobic maintenance phase. The aerobic capacity training phase consisted of low intensity training sessions with a training HR target zone of 140–170 bpm. The anaerobic capacity phase was focussed on high intensity training sessions with a training HR of 170–180 bpm. The power and speed phase included training sessions of higher intensity with moderate recovery periods and no targeted HR zones. The taper training phase consisted of varied intensity training sessions with reduced training volume and no specific HR target zones (Table 2).

The initial training session of each week (*i.e.*, 6:00 a.m. Monday) had an aerobic capacity focus, which did not vary throughout the 14-week monitoring period. The training volume was not controlled for this swim session, however the total swimming distance ranged between 3.5 and 4.5 km during the 14-week monitoring period.

The Monday p.m. (3:00 p.m.) training session was considered the main assessment for the week and the coach used this training session as a gauge of progressive training adaptation. This controlled session was standardised with a consistent volume and swimming activities completed each week across the 14-week monitoring period. The main swimming activities consisted of swimming a total distance of 2.4 km with a 100 m recovery swim (2.5 km total) and was considered a high intensity workload. Volume and intensity of the Monday p.m. training session were consistent throughout the monitoring period, however the training focus shifted according to the training phase (Table 2).

### 2.3. Procedures

All HR recordings (overnight and resting afternoon) were analysed using Kubios HRV software (v2.1, University of Kuopio, Kuopio, Finland). To ensure a consistent analysis for all athletes, HRV was calculated from the most stable (visually inspected for HR change of less than 10 beats) five minute period (*i.e.*, between 3:00 a.m. and 4:00 a.m. during night time sleep and the last 5 min of the afternoon recording. Ectopic beats and artefact (<5%) were identified and corrected using Kubios’ in-built cubic splice interpolation. Along with mean HR, analysis of HRV included time-domain (root mean square of the successive differences (RMSSD)), frequency domain (high frequency normalized units (HFnu)) and non-linear indices (standard deviation of instantaneous RR variability (SD1) and short term fractal scaling exponent (α1)) as previously described [22]. Prior to the commencement of each training session (Monday a.m., Monday p.m. and Tuesday a.m.), saliva samples were obtained from each athlete using an IPRO oral fluid collector (OFC; IPRO Interactive, Oxfordshire, UK). The OFC contains a volume adequacy indicator with a colour change evident (white to dark blue) once 0.5 mL of saliva has been collected. Immediately after collection, each saliva sample was assessed for sIgA and sAA concentrations using an IPRO lateral flow device (IPRO Interactive, Oxfordshire, UK) point of care system as previously described [23]. This method of saliva analysis has previously been validated against ELISA analysis (*R*^2^ = 0.78) [24].

### 2.4. Data and Statistical Analysis

Comparisons of athlete responses were conducted across two time periods—Monday a.m. to Monday p.m. and Monday a.m. to Tuesday a.m. Acute responses were examined from Monday a.m. to Monday p.m. to assess the influence of a light training session along with the normal daily influences on HRV, sIgA and sAA. To further examine acute training responses, changes in HRV, sIgA and sAA were examined from Monday a.m. to Tuesday a.m. to identify the influence of a heavy training session, while also accounting for the impact of normal daily influences and a light training session. These comparisons were incorporated to eliminate the impact of diurnal variation and to focus on the effect of high intensity training and its recovery. Acute changes for HRV, sIgA and sAA are presented in relative units (*i.e.*, Monday p.m.—Monday a.m./Monday a.m. × 100 = % change). Weekly comparisons were conducted after transforming the acute change (*i.e.*, +100) and were expressed relative to week one responses.

The practical significance of weekly changes during the monitoring period were assessed via magnitude-based inferences using a downloaded spread sheet [25]. Using standardised differences in means (*i.e.*, Effect Size (ES)) the weekly change was examined with ES threshold values established as small (0.2), moderate (0.6), large (1.2) and very large (2.0) [26]. Magnitude-based inferences were performed using the smallest worthwhile change calculated as 0.2 of the between-subjects standard deviation. Potentially significant positive, trivial or negative changes were identified qualitatively as: almost certainly (>99.5%); very likely (99.5%–95%); likely (95%–75%); possibly (75%–25%); unlikely (25%–5%); very unlikely (5%–0.5%); and almost certainly not (<0.5%). In line with previous research, the true difference was deemed ‘unclear’ if the chances of having positive and negative changes were both greater than 5% [27].

## 3. Results

### 3.1. Change from Monday a.m. to Monday p.m.

The percentage change for mean HR at Week 1 was 26.88% (Table 3). Compared to Week 1, changes in mean HR were possibly greater at Week 6, with all other changes unclear during the 14-week monitoring period (Table 3).

The percentage change for HF(nu) at Week 1 was −21.61% (Table 3). Compared to Week 1, changes in HF(nu) were possibly smaller (less negative) at Week 7 and likely smaller (less negative) at Week 10 and Week 13 (Table 3). In contrast, the HF(nu) change was possibly greater (more negative) at Week 6 (Table 3).

The percentage change for SD1 was −26.71% at Week 1 (Table 3). Changes in SD1 compared to Week 1 were likely smaller (less negative) at Week 7 and Week 9 while also possibly smaller (less negative) at Week 13 (Table 3). In contrast, changes in SD1 were possibly greater (more negative) at Week 3 and Week 6, with all other changes trivial or unclear (Table 3).

The percentage change for α1 was 44.64% at Week 1 (Table 3). Compared to Week 1, changes in α1 were unclear except for Weeks 6 and Week 12 which were possibly greater, and Week 7 which was possibly smaller (Table 3).

**Figure 1:**
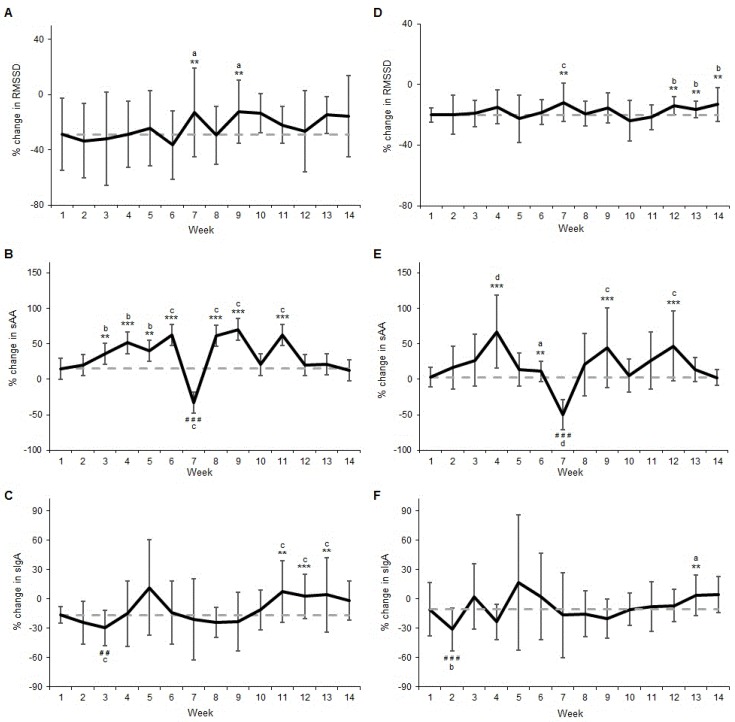
Percentage change (±SD) from Monday a.m. to Monday p.m. for: (**a**) RMSSD (**b**) sAA; (**c**) sIgA; and percentage change from Monday a.m. to Tuesday a.m. for (**d**) RMSSD (**e**) sAA; and (**f**) sIgA over the 14-week monitoring period. SD = Standard Deviation, RMSSD = square root of the mean squared difference of successive RR intervals, sAA = salivary alpha amylase, sIgA = salivary immunoglobulin A. Dashed line represents Week 1 response for comparison. ** likely greater; *** very likely greater; # # likely lower; # # # very likely lower; a indicates small effect size; b indicates moderate effect size; c indicates large effect sizeA.

The percentage change for RMSSD at Week 1 was −28.83% (Figure 1A). Changes for RMSSD were likely smaller (less negative) at Week 7 (78/18/3, ES = 0.40) and Week 9 (83/14/2, ES = 0.44). All other RMSSD changes over the 14-week monitoring period were trivial or unclear (Figure 1A).

For sAA, the percentage change at Week 1 was 15.00% (Figure 1B). Weekly changes for sAA were likely greater for Week 3 (91/6/2, ES = 0.77) and Week 5 (89/7/4, ES = 0.87) while also very likely greater for Weeks 4 (98/2/1, ES = 1.13), 6 (96/2/1, ES = 1.35), 8 (98/1/1, ES = 1.32), 9 (99/1/0, ES = 1.47) and 11 (98/1/0, ES1.35) during the 14-week monitoring period (Figure 1B). In contrast, the sAA change was very likely less (more negative) for Week 7 (0/0/99, ES = −2.46) (Figure 1B).

The percentage change for sIgA was −16.43% at Week 1 (Figure 1C). Weekly changes were mostly trivial or unclear for sIgA when compared to the Week 1 response (Figure 1C). However, sIgA change was likely greater for Week 11 (93/3/4, ES = 1.78), Week 12 (98/1/1, ES = 1.57) and Week 13 (94/4/2, ES = 1.22) (Figure 1C). In contrast, the sIgA change was very likely less for Week 3 (3/3/94, ES = −1.63) (Figure 1C).

### 3.2. Change from Monday a.m. to Tuesday a.m.

The percentage change for mean HR was 5.45% at Week 1 (Table 4) with all changes during the 14-week monitoring period being trivial or unclear (Table 4).

The percentage change at Week 1 was −2.10% for HF(nu) (Table 4) with all changes being trivial or unclear during the 14-week monitoring period (Table 4).

For SD1, the percentage change was −18.67% at Week 1 (Table 4). Changes for SD1 were likely smaller (less negative) for Week 12 and Week 14 (Table 4). In contrast, the change in SD1 at Week 10 was very likely greater (more negative), with all other changes unclear during the 14-week monitoring period (Table 4).

The percentage change for α1 was 12.34% at Week 1 (Table 4). Compared to Week 1, changes in α1 were unclear except for Weeks 8, 11 and 12, which were likely and possibly greater, respectively (Table 4).

The percentage change for RMSSD at Week 1 was −20.09% (Figure 1D). Changes in RMSSD were likely smaller (less negative) for Week 7 (90/5/5, ES = 1.30), Week 12 (93/4/2, ES = 1.01), Week 13 (83/11/5, ES = 0.63) and Week 14 (92/5/3, ES = 1.11) (Figure 1D). All other changes during the 14-week monitoring period were trivial or unclear (Figure 1D).

The percentage change at Week 1 for sAA was 2.95% (Figure 1E). The change in sAA was likely greater at Week 6 (83/14/3, ES = 0.49), while also very likely greater for Week 4 (99/0/0, ES = 2.70), Week 9 (99/1/0, ES = 1.79) and Week 12 (96/2/2, ES = 1.91) (Figure 1E). In contrast, the change in sAA was very likely less (more negative) at Week 7 (0/0/99, ES = −4.69) (Figure 1E). All other changes in sAA were unclear during the 14-week monitoring period (Figure 1E).

The percentage change for sIgA was −10.87% at Week 1 (Figure 1F). The majority of changes for sIgA were trivial or unclear during the 14-week monitoring period (Figure 1F). However, sIgA was likely greater for Week 13 (86/11/2, ES = 0.53) while also very likely less for Week 2 (0/1/99, ES = −0.85) (Figure 1F).

## 4. Discussion

For the first time, the present study examined cardiac autonomic activity and salivary responses in relation to normal daily influences (Monday a.m. to Monday p.m.) while also examining acute (<24 h, Monday a.m. to Tuesday a.m.) responses to a standardised swimming, training bout. Additionally, this study highlighted the chronic training responses of acute changes in HRV, sIgA and sAA over a 14-week monitoring period, leading into a major international competition.

### 4.1. Change from Monday a.m. to Monday p.m.

The current study demonstrated the acute increases of HR, α1 and sAA with corresponding decreases in RMSSD, HF(nu), SD1 and sIgA from Monday morning to Monday afternoon.

The increase in mean HR and α1 along with the reciprocal decrease in RMSSD, HF(nu) and SD1 was likely a result of the circadian changes in cardiac autonomic activity and normal daily activities [28,29]. Earlier studies have reported that parasympathetic activity is typically elevated in the morning while sympathetic modulation increases throughout the day as a result of circadian changes in neural discharge and daily activities [28,29]. However, these prior studies were conducted at rest and did not include the impact of daily activities. While the current athletes did not partake in any strenuous physical activity during the day, all athletes were studying at high school or university and as such did not rest all day between training sessions. For the current study, athletes undertook a morning training session that likely increased HR and reduced HRV, though this change was likely to be short-acting due to the low intensity nature of the training session. A previous study has shown that cardiac parasympathetic activity typically returns to baseline within 15 min following low intensity aerobic exercise [7] with the current daily change in HRV likely to be a normal result of daily activities and autonomic nervous system circadian rhythm.

Similar to the change in HRV mentioned above, there was also a consistent increase in sAA from Monday morning to Monday afternoon for most weeks of the 14-week monitoring period. As all training sessions on Monday morning were completed at a low intensity with acute elevations in sAA reported to be short [30,31], the increase in sAA during Monday was likely due to the typical diurnal pattern in this measure. Through examination of hourly levels of sAA in 76 healthy adults (44 women and 32 men), Nater and colleagues [32] identified an average daily increase of approximately 35%. A slightly lower daily increase in sAA (average increase of 18%) was observed for athletes in the current study, suggesting that athletes may display a lower than normal daily variance in sAA. However, of note was the significantly greater sAA increases for most weeks between Week 3 and 11 (average increase of 55%). This response was greater than the observed average daily increase reported by Nater and colleagues [32] and may represent an accumulated stress response from weekly training for athletes. Remarkably, none of the HRV parameters exhibited this trend for Weeks 3–11 indicating that HRV and sAA may reflect different training responses with sAA potentially more sensitive to the combined stress of training and daily activities. For examples, during Week 6, athletes experienced a greater increase in HR and α1 with corresponding decrease in HF(nu) and SD1 suggesting that athletes were more stimulated over the day. No noticeable changes in training volume or intensity were noted for athletes, with these greater than normal (Week 1) responses potentially a result of an increase in daily activities.

Regardless, the reported Monday a.m. to p.m. changes in HRV and sAA in the current study provide coaches and athletes with normative data to assist in the monitoring of training.

Another key observation from the present study was the negative change in sAA from Monday morning to Monday afternoon at the beginning of Week 7. This assessment was done following a competitive weekend of races with a subsequent elevation in sAA levels Monday morning and a negative change by Monday afternoon. A recent study has shown that elite level competition produces a sustained increase in sAA for up to 48 h after a national swimming grand prix competition similar to that in the current study [21]. Increased levels of cortisol have also been reported after a rugby league match [33] further supporting that high-level competition induces a prolonged stress response, which may influence subsequent training and performance. The current study extends these previous findings and indicates that competition maintains stress responses (*i.e.*, sAA) for some time after the event. Further, this stress response was not altered by a light training session soon after the competition (<24 h) and highlights the need for rest to return athletes to pre competition homeostasis [34].

Accompanying this heightened stress response at the beginning of Week 7, there were smaller a.m. to p.m. responses for HF(nu), SD1 and RMSSD (*i.e.*, smaller negative changes) compared to Week 1. This Week-7 response reflected heightened sympathetic and/or attenuated parasympathetic modulations (*i.e.*, HFnu, SD1 and RMSSD) at Monday a.m. from the lasting effects of the weekend competition, which were altered to a lesser degree during the day. Previous research has highlighted the sustained impact of competition on HRV with a significant reduction in HRV for 48 h after a rugby league match [22] and following a national swimming grand prix [21] being reported. The current study provides further evidence of important physiological changes (*i.e.*, HRV and sAA) in response to severe effort (competition) [21,22,33]. Interestingly, changes in mean HR and the salivary marker of immune function (sIgA) were not different at Week 7 compared to Week 1 indicating that these measures may be insensitive to the stress responses in the days following competition. Subsequently, the monitoring of HRV (HFnu, SD1, and RMSSD) and sAA may provide sound indicators of stress response to training and high level competition for coaches to better manage training workloads of their athletes [21].

### 4.2. Change from Monday a.m. to Tuesday a.m.

Examining the change in HRV, sIgA and sAA from Monday morning to Tuesday morning highlighted a number of key findings. Similar to the observed change for Monday a.m. to Monday p.m. responses, there were comparable Monday a.m. to Tuesday a.m. changes in mean HR and HRV for most weeks over the monitoring period. The a.m. to a.m. changes though were of a smaller magnitude compared to the a.m. to p.m. changes (see Table 3 and Table 4) and likely reflected a similar morning level of cardiac autonomic activity and recovery from the p.m. training session. However, at the beginning of Week 7 (post-competition weekend) there was a greater a.m. to a.m. response (*i.e.*, smaller absolute change) in RMSSD compared to Week 1 that was accompanied by changes in sAA. As described before, the Week 7 response was likely a result of lower HRV and parasympathetic modulations (RMSSD) and greater stress levels (sAA) at Monday a.m. following the competition weekend. Importantly, the overnight response for HRV and sAA in Week 7 did not appear to be altered as a result of the p.m. training session and further highlights the acute effects of training [7] and importance of rest to restore homeostatic balance [34].

Another significant finding from the current study was the greater a.m. to a.m. response for RMSSD towards the end of the monitoring period (*i.e.*, Weeks 12–14). Earlier studies have shown that cardiac autonomic activity may “rebound” from acute training sessions to levels above pre-training, resting values [25,35], similar to that observed in the current study. Interestingly, this response was not evident in the early stages of the monitoring period (Weeks 1–11), suggesting the rebound in cardiac vagal activity observed in Weeks 12–14 was a result of the reduction in training load during the taper training phase. Earlier studies have reported increases in RMSSD during periods of reduced training load [36,37] with this increase related to gains in performance [36]. The current study extends these previous results and indicates that cardiac parasympathetic activity increases in response to training, during periods of reduced training load. This greater training response for RMSSD may further indicate an increased readiness for athlete’s to deal with stressors. Future research may identify whether assessing changes in HRV in response to a standardized training bout, as opposed to measuring the absolute change in HRV over time, provides a more detailed understanding of training responses in elite athlete populations.

Curiously, there were likely greater increase in sAA for some Weeks (4, 6, 9 and 12) but not others. There was no apparent trend for these responses and they did not coincide with commencement/end of training phases or with HRV measures. Speculatively, these results may indicate aftereffects of the high intensity training bout on Monday afternoon in combination with the stress responses associated with daily activities previously discussed however, this response was inconsistent. Further investigation may elaborate on the mechanisms and practical implications for such responses.

Lastly, a greater increase in sIgA was noted for Week 13, a taper phase. Earlier studies have reported decreases in sIgA following high intensity exercise bouts, which may alter immune function for up to 72 h and increase an athlete’s susceptibility to illness [38,39,40]. However, previous research has also observed increases in sIgA following various forms of high intensity exercise [41,42,43]. In the current study the increase in sIgA was reported at 14 h post exercise and as such may not have been the peak value. Therefore the current results highlight a prolonged increase in sIgA, contrary to previous studies [44,45]. No athletes reported any signs of illness during the taper period suggesting that the high intensity training session had no negative influence on the athlete’s immune function in the lead up to competition. Additionally, the greater response for sIgA was only observed in Week 13, with greater responses also noted for RMSSD in the week before, during and after sIgA increase (*i.e.*, Weeks 12–14). Subsequently, the reduction in training load, typical during a taper period, may result in heightened parasympathetic modulations along with increased immune function, and provide an appropriate strategy to minimize fatigue and optimize the health status of athletes prior to competition. Further, RMSSD may provide a more sensitive and early indication of the positive training responses during periods of reduced training load. Future studies are encouraged to examine performance measures to substantiate the potential application of sIgA monitoring during long-term training in athletic populations.

While the current results are based on a small sample size (*n* = 8), numbers of this size are typical of this athlete population, and common in research using elite athlete populations [21,46,47]. Furthermore, statistical analysis was adjusted accordingly with data analysed using magnitude-based inferences to account for individual variations in HRV and saliva variables and to best examine the practical significance of the data when investigating smaller populations. Additionally, the use of nocturnal HRV measures may have limited the current results. Seated HRV measurements have been shown to potentially limit the effect of HRV saturation and as such may provide more beneficial information when compared to nocturnal HRV measures.

## 5. Conclusions

This unique study has documented the impact of daily activities on HRV and saliva (sIgA and sAA) responses with and without a standardized training bout repeated over a 14-week monitoring period. The current study has identified that cardiac autonomic activity and salivary markers of immune function and stress responded consistently to daily activities and training, likely a result of the effective training and recovery prescription. Additionally, the study highlighted important physiological changes (*i.e.*, HRV and sAA) in response to competition and subsequent rest/recovery. Lastly, HRV appears to be more a sensitive indicator of positive training responses during periods of reduced training load when compared with sIgA. Gaining a better understanding of how these physiological markers respond to various forms of training intensity will lead to an improved understanding of training responses and recovery cycles along with highlighting the early signs of positive and negative training responses in elite athlete populations.

## Figures and Tables

**Table 1:** Athlete Characteristics.

Athlete	Sex	Age (years)	Impairment	Athlete (Classification) ^a^	National Experience (years) ^b^
Athlete 1	F	20	Intellectual	S14	2
Athlete 2	M	29	Amputee	SM10	11
Athlete 3	M	17	Vision	S13	1
Athlete 4	F	15	Cerebral Palsy	S8	1
Athlete 5	M	24	Amputee	S8	6
Athlete 6	M	27	Neuromuscular	S10	11
Athlete 7	M	16	Amputee	S9	1
Athlete 8	M	19	Vision	S13	2

^a^ IPC Classification code; ^b^ Years competing as part of the national Paralympic swimming squad.

**Table 2:** Average weekly kilometres completed during the 14-week monitoring period.

Training Phase	Week														
	1	2	3	4	5	6	7	8	9	10	11	12	13	14	15
Aerobic Maintenance	28	-	-	-	-	-	-	-	-	-	-	-	-	-	-
Aerobic Capacity	-	34	43	47	48	-	-	-	-	-	-	-	-	-	-
Anaerobic Capacity	-	-	-	-	-	45	48	48	45	47	-	-	-	-	-
Power and Speed	-	-	-	-	-	-	-	-	-	-	46	35	-	-	-
Taper	-	-	-	-	-	-	-	-	-	-	-	-	28	29	-
Competition						X			X				X		X
Swimming Test			X								X				

X indicates competition or swimming test.

**Table 3:** Monday morning to Tuesday morning change (%) and qualitative inferences for heart rate variability over the 14-week monitoring period.

Change from Monday a.m. to Monday p.m.														
Week	1	2	3	4	5	6	7	8	9	10	11	12	13	14
HR	26.88	30.71	32.09	28.12	27.21	35.85	20.74	23.02	23.33	21.90	31.55	30.47	19.80	17.54
(±SD)	(±23.5)	(±29.8)	(±31.5)	(±20.0)	(±20.9)	(±26.4)	(±14.6)	(±13.8)	(±18.0)	(±17.4)	(±19.8)	(±21.0)	(±14.2)	(±11.4)
ES		0.12	0.14	0.07	0.02	0.33	−0.21	−0.11	−0.12	−0.17	0.20	0.15	−0.25	−0.33
QI		Unclear	Unclear	Unclear	Unclear	Possibly +ve	Unclear	Unclear	Unclear	Unclear	Unclear	Unclear	Unclear	Unclear
		37/56/7	45/38/17	32/51/16	25/56/19	72/24/4	12/34/54	16/43/41	21/36/43	7/44/49	54/39/7	46/45/9	12/30/58	6/24/70
HF(nu)	−21.61	−13.03	−26.67	−22.27	−22.75	−24.32	−2.84	−7.31	−22.62	−10.00	−18.58	−14.11	2.43	−5.68
(±SD)	(±57.2)	(±53.7)	(±46.1)	(±44.0)	(±35.4)	(±70.2)	(±53.1)	(±86.7)	(±31.9)	(±38.7)	(±33.2)	(±63.3)	(±59.9)	(±74.3)
ES		0.08	−0.03	0.05	0.11	−0.32	0.33	−0.07	0.15	0.34	0.18	0.11	0.39	0.25
QI		Unclear	Unclear	Trivial	Unclear	Possibly −ve	Possibly +ve	Unclear	Unclear	Likely +ve	Unclear	Unclear	Likely +ve	Unclear
		33/56/12	14/65/21	11/87/2	41/45/15	4/25/71	71/24/5	16/52/32	46/35/18	76/22/3	51/44/6	37/54/8	83/16/1	60/31/9
SD1	−26.71	−32.74	−32.05	−28.88	−22.10	−36.53	−13.35	−33.77	−10.05	−12.43	−19.65	−22.92	−19.53	−13.65
(±SD)	(±26.6)	(±26.7)	(±33.7)	(±24.1)	(±25.9)	(±24.8)	(±20.2)	(±25.8)	(±21.5)	(±14.2)	(±14.9)	(±27.2)	(±15.9)	(±28.0)
ES		−0.17	−0.23	−0.01	0.16	−0.23	0.38	−0.13	0.44	0.42	0.28	0.16	0.28	0.35
QI		Trivial	Possibly −ve	Unclear	Unclear	Possibly −ve	Likely +ve	Unclear	Likely +ve	Unclear	Unclear	Unclear	Possibly +ve	Unclear
		1/51/47	5/35/59	12/73/14	48/39/13	1/33/66	77/19/4	7/54/40	79/17/4	74/19/7	61/28/11	48/33/18	66/29/5	68/23/9
α1	44.64	38.18	50.24	58.37	19.85	65.13	33.21	57.79	35.43	29.47	49.97	71.56	33.13	33.07
(±SD)	(±55.4)	(±53.6)	(±47.8)	(±60.7)	(±34.6)	(±85.3)	(±84.0)	(±78.8 )	(±57.2)	(±55.3)	(±61.9)	(±91.4)	(±87.5)	(±53.6)
ES		−0.08	0.13	0.21	−0.35	0.21	−0.32	0.10	−0.13	−0.27	0.08	0.28	−0.31	−0.17
QI		Unclear	Unclear	Unclear	Unclear	Possibly +ve	Possibly −ve	Unclear	Unclear	Unclear	Unclear	Possibly +ve	Unclear	Unclear
		7/64/28	43/46/11	55/39/6	8/24/69	57/39/5	5/26/69	38/48/15	17/39/44	11/28/61	36/47/17	68/29/3	8/26/66	9/41/49

Heart rate = HR; High frequency normalised units = HF(nu); standard deviation of instantaneous RR variability = SD1; short term fractal scaling exponent = α1; SD = Standard Deviation; ES = Effect Size; QI = Qualitative Inference; +ve = Positive; −ve = Negative.

**Table 4:** Monday morning to Tuesday morning change (%) and qualitative inferences for heart rate variability over the 14-week monitoring period.

Change from Monday a.m. to Tuesday a.m.														
Week	1	2	3	4	5	6	7	8	9	10	11	12	13	14
HR	5.45	6.86	6.16	7.73	3.98	9.44	7.75	7.10	6.29	9.26	8.34	6.43	3.56	5.28
(±SD)	(±12.3)	(±8.5)	(±9.9)	(±7.6)	(±13.9)	(±10.7)	(±16.1)	(±9.1)	(±12.9)	(±7.6)	(±8.5)	(±10.0)	(±6.6)	(±5.8)
ES		0.12	0.07	0.18	−0.12	0.28	0.14	0.13	0.06	0.29	0.22	0.08	−0.10	0.03
QI		Trivial	Unclear	Unclear	Unclear	Unclear	Unclear	Unclear	Unclear	Unclear	Unclear	Unclear	Unclear	Unclear
		34/63/3	39/33/28	51/40/9	20/37/43	60/26/14	47/29/25	46/30/24	40/29/31	62/27/11	54/28/18	30/62/8	25/33/42	31/43/26
HF(nu)	−2.10	−8.38	−11.94	−5.20	−7.40	11.80	18.43	−13.91	−6.62	−19.42	−18.04	−15.17	7.55	11.17
(±SD)	(±40.3)	(±35.4)	(±34.5)	(±39.8)	(±35.6)	(±40.4)	(±55.4)	(±22.4)	(±39.5)	(±25.9)	(±20.3)	(±29.3)	(±46.4)	(±79.8)
ES		−0.03	−0.07	0.02	−0.04	0.26	0.33	−0.06	−0.02	−0.21	−0.14	−0.14	0.21	0.10
QI		Unclear	Unclear	Unclear	Unclear	Unclear	Unclear	Unclear	Unclear	Unclear	Unclear	Trivial	Unclear	Unclear
		25/44/31	18/47/34	28/49/23	34/26/39	60/30/10	69/24/6	20/47/33	34/28/38	9/37/55	11/46/43	3/59/38	53/27/20	41/37/22
SD1	−18.67	−21.80	−19.03	−14.80	−17.52	−18.08	−2.83	−19.83	−15.03	−23.81	−20.00	−13.11	−16.04	−11.61
(±SD)	(±6.4)	(±11.9)	(±8.7)	(±11.1)	(±25.5)	(±8.1)	(±26.6)	(±8.7)	(±9.6)	(±13.3)	(±8.8)	(±6.5)	(±5.5)	(±10.8)
ES		−0.53	−0.08	0.47	−0.25	0.06	1.70	−0.19	0.46	−0.86	−0.22	0.74	0.36	0.89
QI		Unclear	Unclear	Unclear	Unclear	Unclear	Unclear	Unclear	Unclear	Likely −ve	Unclear	Likely +ve	Unclear	Likely +ve
		10/15/74	29/30/41	67/16/17	33/14/53	39/32/29	90/4/6	23/26/51	70/17/13	5/8/88	16/30/54	88/8/4	67/22/11	92/6/3
α1	12.34	4.18	19.60	18.61	9.45	22.15	4.08	46.60	4.67	17.58	29.94	22.26	11.40	20.91
(±SD)	(±41.5)	(±33.7)	(±29.1)	(±25.2)	(±39.3)	(±58.2)	(±49.0)	(±43.3)	(±24.3)	(±28.3)	(±29.8)	(±40.3)	(±23.7)	(±56.1)
ES		−0.17	0.23	0.23	−0.09	0.14	−0.28	0.75	−0.12	0.20	0.47	0.25	0.07	0.14
QI		Unclear	Unclear	Unclear	Unclear	Unclear	Unclear	Likely +ve	Unclear	Unclear	Likely +ve	Possibly +ve	Unclear	Unclear
		7/45/48	56/31/13	56/29/15	31/25/44	47/31/22	10/28/62	94/5/1	24/31/45	54/37/10	80/16/4	65/33/2	38/38/25	46/34/20

Heart rate = HR; High frequency normalised units = HF(nu); standard deviation of instantaneous RR variability = SD1; short term fractal scaling exponent = α1; SD = Standard Deviation; ES = Effect Size; QI = Qualitative Inference; +ve = Positive; −ve = Negative.
